# Analysis of the Complete Genome Sequence of* Bacillus atrophaeus* GQJK17 Reveals Its Biocontrol Characteristics as a Plant Growth-Promoting Rhizobacterium

**DOI:** 10.1155/2018/9473542

**Published:** 2018-06-26

**Authors:** Jinjin Ma, Chengqiang Wang, Haide Wang, Kai Liu, Tongrui Zhang, Liangtong Yao, Zhou Zhao, Binghai Du, Yanqin Ding

**Affiliations:** College of Life Sciences, National Engineering Laboratory for Efficient Utilization of Soil and Fertilizer Resources, Shandong Key Laboratory of Agricultural Microbiology, Shandong Agricultural University, Tai'an, China

## Abstract

*Bacillus atrophaeus* GQJK17 was isolated from the rhizosphere of* Lycium barbarum* L. in China, which was shown to be a plant growth-promoting rhizobacterium as a new biological agent against pathogenic fungi and gram-positive bacteria. We present its biological characteristics and complete genome sequence, which contains a 4,325,818 bp circular chromosome with 4,181 coding DNA sequences and a G+C content of 43.3%. A genome analysis revealed a total of 8 candidate gene clusters for producing antimicrobial secondary metabolites, including surfactin, bacillaene, fengycin, and bacillibactin. Some other antimicrobial and plant growth-promoting genes were also discovered. Our results provide insights into the genetic and biological basis of* B. atrophaeus* strains as a biocontrol agent for application in agriculture.

## 1. Introduction

In recent years, the yield and quality of many medicinal plants, vegetables, fruits, and crops have decreased because of plant diseases caused by soil-borne pathogens [1–4]. Moreover, a large number of chemical pesticides and fertilizers have been used in agriculture that further caused quality reduction of agricultural products [5], pathogen resistance to chemicals [1], and environmental pollution [6]. Plant growth-promoting rhizobacteria (PGPR) are a group of strains that localize in the plant rhizosphere and play an important role in preventing and controlling soil-borne diseases [7], promoting plant growth and development [8, 9], enhancing stress tolerance [10], and regulating and improving the rhizosphere soil environment [11–13].* Bacillus* species are an important group of PGPR, and some of them have been widely used in agriculture as biocontrol agents [14–16].

*Bacillus atrophaeus* as a group of useful bacterium has been studied in many aspects.* B. atrophaeus* was verified to be a known biomolecule producer [17], which could produce bacteriocin [18], bioactive compounds [19], and biosurfactant proteins [20].* B. atrophaeus* is also an important group of PGPR.* B. atrophaeus* M-35 was recognized as a PGPR member, and it was previously identified to effectively inhibit potato dry rot and rhizome rot of ginger caused by* Fusarium* species [21, 22].* B. atrophaeus* also exhibits a strong inhibitory effect against poplar anthracnose caused by a predominant fungus,* Colletotrichum gloeosporioides* [23].* B. atrophaeus* CAB-1 was reported to display a high inhibitory activity against various fungal pathogens and was capable of suppressing cucumber powdery mildew and tomato gray mold [24]. Moreover,* B. atrophaeus* had an extraordinary activity in root colonization and crop protection [25], and it was verified to promote the growth of* Zea mays L.* and* Solanum lycopersicum* [26]. However, the biocontrol mechanisms of* B. atrophaeus* species as PGPR have not been well characterized to date.

The goji berries produced by* Lycium barbarum* L. have nutritional health and medicinal value [27, 28] because of the contained components, such as* Lycium barbarum* L. polysaccharides and betaine [29, 30]. They can enhance human immunity, regulate blood fat, lower blood pressure, inhibit the growth and mutation of cancer cells, resist radiation, and so on [31–33]. So, the cultivated land for* Lycium barbarum* L. has been increased year by year, especially in China. With the continuous expansion of planting areas for* Lycium barbarum* L. and the continuous planting activity year by year, a variety of fungal soil-borne diseases are arising and seriously affecting the yield and quality of* Lycium barbarum* L. Root rot is one of the most important diseases of* Lycium barbarum* L. and* Fusarium *spp. (e.g.,* F. solani*) are the main pathogens [34].

Strain GQJK17 was isolated from the rhizosphere of* Lycium barbarum* L. in Ningxia, China, and identified to be a plant growth-promoting rhizobacterium aimed at the root rot of* Lycium barbarum* L. It was identified to be* B. atrophaeus* and has the most significant inhibition effect on the root rot pathogen* F. solani* among all the selected strains. To further study the genetic basis and molecular mechanism of its biocontrol ability, we performed the complete genome sequencing and annotation. The secondary metabolic gene clusters for pathogen resistance and some plant growth-promoting genes are discovered.

## 2. Materials and Methods

### 2.1. Strain Isolation and Property Analysis

Strain GQJK17 was isolated from rhizosphere soil samples of* Lycium barbarum* L. collected from Ningxia, China. All physiological and biochemical tests were performed at 37°C. The colony morphology was determined after 24 h incubation on LB agar medium. Cellular morphology and spore detection were performed by spore staining using 5% malachite green dye and 0.5% fuchsin dye [43] and examined by fluorescence microscopy (Olympus, Japan). Some physiological and biochemical characteristics of GQJK17 were determined as follows. Oxidase activity was determined using 1% solution of tetramethyl-*p*-phenylenediamine [44]. Catalase activity was determined by assessing the production of bubbles after the addition of a drop of 3% H_2_O_2_ [45]. Nitrate reduction, methyl red test (M-R), Voges-Prokauer reaction (V-P), indole production, and carbon utilization were tested using the bacteria microbiochemical identification tube (HOPEBIO, China) [45].

### 2.2. The Phylogenetic Analysis

The genomic DNA of strain GQJK17 was extracted using the genomic DNA kit (TIANGEN, China). The polymerase chain reaction (PCR) was performed as follows: 5 min at 95°C (predegeneration); 30 cycles of 1 min at 94°C (denaturation), 1 min at 56°C (annealing), and 1 min at 72°C (extension); followed by 10 min at 72°C (final extension). The 16S rDNA sequence was obtained using primers 27F (5′-AGAGTTTGATCCTGGCTCAG-3) and 1492R (5′-GGTTACCTTGTTACGACTT-3′) and then analyzed by the National Center for Biotechnology Information (NCBI) (http://www.ncbi.nlm.nih.gov). The neighbor-joining phylogenetic tree was constructed with some species of the genus* Bacillus* based on the 16S rDNA sequences by MEGA 6.0.

### 2.3. The Determination of Antagonistic Properties

The antagonistic experiments were performed as reported [46]. The antifungal activity of strain GQJK17 was tested against* Fusarium solani*.* F. solani* with a diameter of 6 mm was inoculated in the center of a PDA agar plate and cultured at 28°C for one day. Then, strain GQJK17 was inoculated in one side of* F. solani* at a distance of 2 cm and incubated for another 3 to 5 days. After incubation, the inhibition zone was observed. The antibacterial assays of GQJK17 were performed against* Escherichia coli* DH5*α* and* Bacillus subtilis* 168. The precultured strain DH5*α* or 168 was incubated in 5 mL of LB liquid medium at 37°C for 10 h. Then, 1 mL of the culture was mixed with 100 mL of LB semisolid medium (with 1% agar) and poured into a sterile Petri dish. Strain GQJK17 was inoculated on the center of a cooled medium and incubated at 28°C for 24 h.

### 2.4. Medium Optimization

The culture medium of strain GQJK17 was optimized using bean sprouts as the basic medium. The plate counting method was used to estimate the strain growth. The suitable carbon sources (sucrose, glucose, lactose, corn flour, and soluble starch), nitrogen sources (including the organic nitrogen sources: beef extract, peptone, yeast powder, and soybean meal, and the inorganic nitrogen source: (NH_4_)_2_SO_4_, NH_4_NO_3_, NH_4_Cl, and Urea), and inorganic salts (MgSO_4_, CaCO_3_, K_2_HPO_4_, and KH_2_PO_4_) were determined by single factor experiments. The orthogonal test (designed by orthogonal design assistant II V3.1) [47] was used to predict the optimum medium for strain GQJK17.

### 2.5. Genome Sequencing and Annotation

The complete genome of strain GQJK17 was sequenced by the Illumina HiSeq and PacBio platforms. The SMRT Analysis 2.3.0 [48] (https://github.com/PacificBiosciences/SMRT-Analysis/wiki/SMRT-Pipe-Reference-Guide-v2.3.0) was used to assemble the whole genome sequence. The NCBI Prokaryotic Genomes Automatic Annotation Pipeline (PGAAP) was used to perform the gene annotation. The gene functions were further analyzed by BLASTP using five databases (Cluster of Orthologous Groups of proteins: COG, Gene Ontology: GO, Kyoto Encyclopedia of Genes and Genomes: KEGG, Non-Redundant Protein Database: NR, and Swiss-Prot). The carbohydrate-active enzyme analyses of the genome also utilized the Carbohydrate-Active enZYmes Database (CAZy) v.20161020 [49] (http://www.cazy.org/). RepeatMasker (3-3-0, http://www.repeatmasker.org/) was used to predict interspersed repeated sequences, and TRF (4.04, http://tandem.bu.edu/trf/trf.html) was used to search tandem repeats. tRNAscan-SE 1.3.1, rRNAmmer 1.2, and Rfam were used to determine tRNA, rRNA, and sRNA, respectively. The potential secondary metabolic gene clusters were predicted using antiSMASH v.4.0.2 [50]. IslandViewer 4 (http://www.pathogenomics.sfu.ca/islandviewer2/query.php) was used to predict genomic islands (GIs) [51]. The complete and circular genome map was created by Circos v.0.64 [52] (http://www.circos.ca/), including noncoding RNAs and gene function annotations.

## 3. Results

### 3.1. The Isolation and Identification of Strain GQJK17

**Figure 1:**
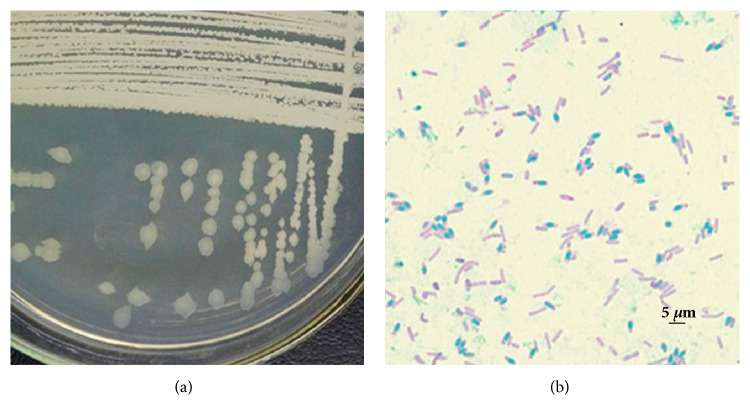
Morphological characteristics of GQJK17. Colony morphology (24 h) (a) and cellular morphology and spore (magnification 10 × 100) (b). GQJK17 was inoculated on LB agar medium and incubated at 37°C for 24 h. Spores were stained with 5% malachite green dye and 0.5% magenta dye.

Strain GQJK17 was isolated from the rhizosphere soil of* Lycium barbarum* L. in Ningxia, China, and cultivated on LB medium at 37°C. The colony morphology of strain GQJK17 is nearly circular, smooth, moist, and milky white after being cultured on LB agar medium for 24 h. The cellular morphology of strain GQJK17 is rod-shaped and strain GQJK17 can produce spores. Its colony and cellular morphology are shown in Figures 1(a) and 1(b). Some physiological and biochemical traits of strain GQJK17 were tested. The properties of oxidase activity, starch hydrolysis, M-R, and indole production are negative, but the catalase activity, citrate utilization, nitrate reduction, and V-P are positive. The carbon utilization experiments showed that strain GQJK17 could utilize mannitol, Arabic candy, sorbitol, and maltose, but not xylose, cellobiose, and lactose (Table 1).

**Figure 2:**
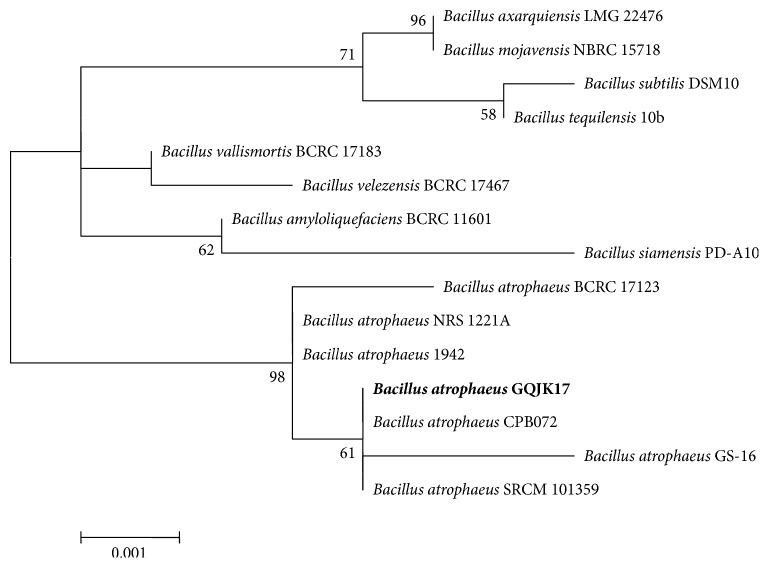
Neighbor-joining phylogenetic tree of* B. atrophaeus* GQJK17 and members of the genus* Bacillus* based on 16S rDNA gene sequences. The phylogenetic tree was constructed using the MEGA 6.0 program and evolutionary distances were computed by the Maximum Likelihood method. Bootstrap values (expressed as percentages of 1000 replications) >50% are indicated at the branch points. The scale bar indicates 0.001 nucleotide substitutions per site.

The phylogenetic analysis of strain GQJK17 based on 16S rDNA sequences was conducted by MEGA 6.0 with related* Bacillus* species (Figure 2) to show the phylogenetic relationships. Strain GQJK17 was successfully clustered to* B. atrophaeus*. Up to now, only four complete genome sequences of this species have been obtained except GQJK17, including strains SRCM101359, 1942, NRS 1221A, and BA59. The closest relative of strain GQJK17 is* B. atrophaeus* SRCM 101359.

### 3.2. The Biocontrol Efficacy of Strain GQJK17

**Figure 3:**
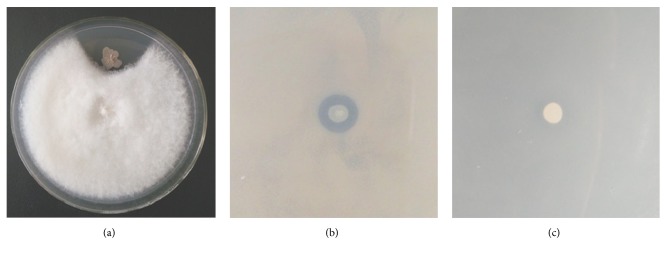
In vitro antagonistic activities of* B. atrophaeus* GQJK17 against* F. solani* (a),* B. subtilis* (b), and* E. coli* (c). The antifungal activity of GQJK17 was tested against* F. solani*. Newly cultivated hyphal plugs of* F. solani* were placed on the center of a PDA plate and incubated for 1 day at 28°C. Then, strain GQJK17 was inoculated onto one side of the plug at a distance of 2 cm and incubated for another 3 days. The antibacterial assays of GQJK17 were performed against* E. coli* and* B. subtilis*. The precultured* E. coli* or* B. subtilis* was incubated in 5 mL of LB liquid medium for 10 h at 37°C. Then, 1 mL of the culture was mixed with 100 mL of LB semisolid medium. Strain GQJK17 was inoculated on the center of the plate and incubated for 1 day at 28°C.

The antagonistic activities of strain GQJK17 against* F. solani*,* B. subtilis* 168, and* E. coli* DH5*α* were tested. In Figure 3, it is shown that strain GQJK17 exhibits antagonistic activity against* F. solani* and* B. subtilis* but no effect on* E. coli* indicating potential applications for controlling some pathogenic fungi and gram-positive bacteria. Among all the isolated* Bacillus* strains, GQJK17 has the most significant inhibitory effect on the root rot pathogen* F. solani* of* Lycium barbarum* L. GQJK17 can adapt to the saline-alkali environment in the isolated area Ningxia. As a PGPR, strain GQJK17 could also produce siderophores and show casein degradation activity (data not show). Thus, GQJK17 was a potential strain to improve plant growth as a biocontrol agent or microbial fertilizer.

### 3.3. The Medium Optimization of Strain GQJK17

To adapt the actual application of strain GQJK17, we also optimized the culture medium of strain GQJK17. The bean sprouts were used as the basic medium, which could provide the necessary nutrients for strain GQJK17 growth and reduce production costs. By single factor experiments, the optimal sources of carbon, organic nitrogen, inorganic nitrogen, and inorganic salt of strain GQJK17 were determined to be glucose, soybean meal, NH_4_NO_3_, and MgSO_4_, respectively. The orthogonal experiments were designed (Supplementary S1) and the optimal medium contained 3% glucose, 1.5% soybean meal, 0.3% NH_4_NO_3_, and 0.3% MgSO_4_. The colony numbers of strain GQJK17 were significantly increased from 3.21 × 10^8^ cfu/mL to 7.98 × 10^9^ cfu/mL using the optimized medium.

### 3.4. Genome Sequence and Genome Features of Strain GQJK17

**Figure 4:**
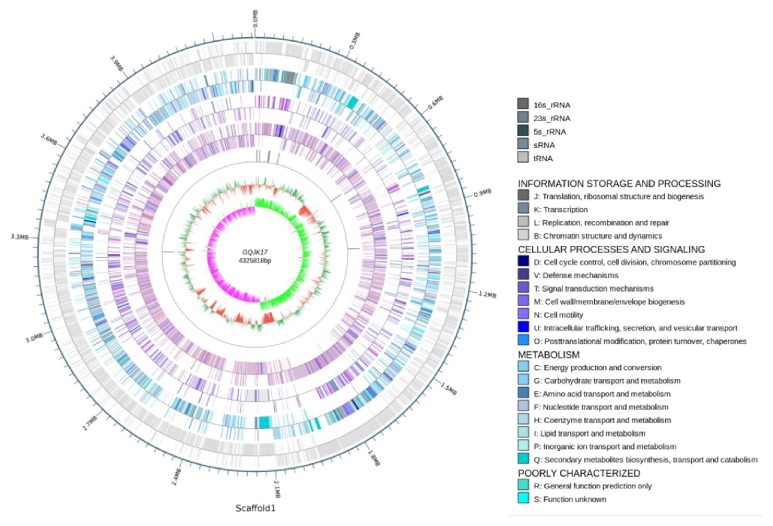
Circular genome map of* B. atrophaeus* GQJK17. From the outside to the center, circle 1: the size of complete genome; circles 2 to 4: the predicted protein-coding genes by using COG, KEGG, and GO databases, respectively, different colors represent different function classifications; circle 5: ncRNA; circle 6: G+C content, with >43.26% G+C in green, with ≤43.26% G+C in red; the inner circle: G+C skew, with G% >C% in peak green, with G% <C% in purple.

A total of 1,364 Mb clean raw data were generated and the coverage of the genome was 307.9×. From the 126,058 subreads, approximately 1,289,931,176 bp were obtained. The genome of* B. atrophaeus* GQJK17 contains a 4,325,818 bp circular chromosome with a G+C content of 43.3%, including 4,015 protein-coding genes, 84 tRNA, 24 rRNA, and 5 ncRNA (Table 2 and Figure 4). No plasmid was found. The whole genome sequence of strain GQJK17 has been deposited in GenBank under the accession number CP022653. There were 3,373 genes that were assigned to the COG databases, accounting for 84.01% among the protein-coding genes (Table 3). Most genes have been annotated; however, 22.53% of the protein-coding genes are poorly characterized and are assigned to the R and S groups. The genes encoding amino acid transport and metabolism, transcription, carbohydrate transport and metabolism, and inorganic ion transport and metabolism account for a large proportion (each more than 6%). Furthermore, the analysis of CAZy showed that 154 genes were related to carbohydrate enzymes. This indicates the better absorption capacity and response ability of this species for amino acids, carbohydrates, and irons in the living environment.

### 3.5. Genetic Basis for Producing Antimicrobial and Plant Growth-Promoting Metabolites

Strain GQJK17 was selected due to its inhibition effects on pathogenic fungi and gram-positive bacteria (Figure 3), which indicated the existence of antimicrobial gene clusters. In the genome of strain GQJK17, a total of 13 secondary metabolic gene clusters were predicted using antiSMASH (v.4.0.2) (Table 4), among them eight gene clusters belonging to nonribosomal peptide synthetases (NRPS) or polyketide synthetases (PKS). These clusters were mainly responsible for biological resistance. The potentially antifungal secondary metabolites were surfactin, fengycin, pelgipeptin, xenocoumacin, bacillomycin, and rhizocticin. Surfactin and pelgipeptin also have antibacterial abilities. There were also gene clusters for antibacterial effects only and the predicted metabolites were bacillaene and anthracimycin. Compared with the other four complete genome sequences of this species, SRCM101359, 1942, NRS 1221A, and BA59, the main gene clusters predicted by antiSMASH (v.4.0.2) for producing antimicrobial secondary metabolites were generally similar. They all contained the biosynthetic gene clusters for surfactin, bacillaene, fengycin, rhizocticin, and bacillomycin. However, the biosynthetic genes of xenocoumacin were only discovered in strains GQJK17 and SRCM101359. In addition, the biosynthetic genes coding for pelgipeptin and anthracimycin only appeared in strain GQJK17. Our findings highlight the evolutionary conservation of molecular genetic mechanism of* B. atrophaeus* strains for biocontrol ability and the specialization of strain GQJK17.

There were four other secondary metabolic gene clusters in strain GQJK17 and the functions of those are still unclear. Interestingly, two gene clusters potentially producing terpenes were identified. Terpenes are a large class of organic compounds produced by some plants, bacteria, and fungi, which may be used as additives in food and cosmetics industries or exhibit antimicrobial or anticarcinogenic properties [53]. Some other antimicrobial-related genes could also be discovered in strain GQJK17, such as the synthetic genes of glucanase, ribonuclease, and proteases.

Moreover, the genome of strain GQJK17 contains many plant growth-promoting genes. One secondary metabolic gene cluster was predicted to produce bacillibactin, which is a kind of strong siderophore that can increase the absorption of ferric ions in soil for plant growth [54]. In addition to the synthetic gene cluster of bacillibactin, strain GQJK17 also harbors other plant growth-promoting genes codifying the production of useful substances, including butanone, phytase, and phosphatase. Other genes are likely involved in cell motility, molecular communication, and environmental responses.

### 3.6. Genomic Islands Analysis

The whole genome sequence of GQJK17 was analyzed by IslandViewer 4, and 14 GIs were discovered (Supplementary S2). Genomic Islands (GIs) are part of the genome sequences presenting the horizontal gene transfer from other species. The GIs in the genome of strain GQJK17 express a variety of proteins mainly involved in glycoside hydrolase, phage proteins, fimbrial proteins, transporters, and regulatory factors. Five phage protein genes are found in GIs, which indicate the previous infection by phages. A polyketide synthase, two glycoside hydrolases were identified, which are related to the antimicrobial activities of GQJK17. Some transcriptional regulators (e.g., LysR family regulator) can regulate the expression genes involved in metabolism, virulence, quorum sensing, and motility [55], and they might be related to the antagonistic properties. Type IV secretion protein Rhs can regulate cell-to-cell contact-dependent competition [56]. Certain Rhs were also reported to have antibacterial activity.

## 4. Discussion

*Lycium barbarum* L. is a significant and commercial crop because of its nutritional and medicinal value [27]. In recent years, due to long-term cultivation and continuous cropping,* Lycium barbarum* L. was increasingly affected by soil-borne diseases in China. Especially, the spread of root rot seriously affected the yield and quality of* Lycium barbarum* L. In this study, we screened out a strain GQJK17 from the rhizosphere of* Lycium barbarum* L. Biocontrol experiments showed that strain GQJK17 could inhibit the pathogen* F. solani* of* Lycium barbarum* L. root rot and also repress the growth of* B. subtilis* 168. Morphological observation and phylogenetic analysis showed that GQJK17 was closely related to* B. atrophaeus* CPB072.* B. atrophaeus* species is a PGPR which has considerable effects on inhibiting some soil-borne diseases and promoting the growth of some plants [22]. The biocontrol characteristics of strain GQJK17 revealed its important roles as a plant growth-promoting rhizobacterium. This strain provides an excellent resource for developing new microbial fertilizers and shows interesting prospects for agricultural applications.

To further study the genetic basis and molecular mechanism of strain GQJK17 as PGPR, we sequenced its complete genome that presented the genetic basis of its biocontrol function and a molecular background for subsequent transformation. Eight secondary metabolic gene clusters were found out, which might be responsible for its function as a new biocontrol agent. Some other plant growth-promoting genes for producing many useful substances were also found in strain GQJK17. Comparing the genome sequence of strain GQJK17 with the other three complete genome sequences of this species, the main gene clusters for producing antimicrobial secondary metabolites were generally similar. Our findings further highlight the evolutionary conservation of* B. atrophaeus* species for biocontrol ability as PGPR.

## Figures and Tables

**Table 1:** Characteristics of physiology and biochemistry of GQJK17.

Properties	GQJK17	Properties	GQJK17
Oxidase	-	Mannitol utilization	+
Catalase	+	Xylose utilization	-
Citrate utilization	+	Cellobiose utilization	-
Starch hydrolysis	+	Arabic candy utilization	+
Nitrate reduction	+	Sorbitol utilization	+
methyl red (M-R)	-	Maltose utilization	+
Voges-Prokauer (V-P)	+	Lactose utilization	-
Indole production	-		

**Table 2:** The general genome feature of *B. atrophaeus* GQJK17.

Feature	Value
Genome size (bp)	4,325,818
G+C content (%)	43.3
Total number of genes	4,294
Total size of protein-coding genes (bp)	3,829,380
Protein-coding genes	4015
Average CDS size (bp)	916
rRNA number	24
tRNA number	84
ncRNA number	5
Pseudo genes (total)	166

**Table 3:** COG categories of* B. atrophaeus* GQJK17.

COG code	Description	Number	Proportion
B	Chromatin structure and dynamics	1	0.03%
C	Energy production and conversion	179	5.31%
D	Cell cycle control, cell division, chromosome partitioning	35	1.04%
E	Amino acid transport and metabolism	340	10.08%
F	Nucleotide transport and metabolism	78	2.31%
G	Carbohydrate transport and metabolism	260	7.71%
H	Coenzyme transport and metabolism	126	3.74%
I	Lipid transport and metabolism	112	3.32%
J	Translation, ribosomal structure and biogenesis	162	4.80%
K	Transcription	289	8.57%
L	Replication, recombination and repair	120	3.56%
M	Cell wall/membrane/envelope biogenesis	186	5.51%
N	Cell motility	60	1.78%
O	Posttranslational modification, protein turnover, chaperones	101	2.99%
P	Inorganic ion transport and metabolism	217	6.43%
Q	Secondary metabolites biosynthesis, transport and catabolism	92	2.73%
R	General function prediction only	454	13.46%
S	Function unknown	308	9.13%
T	Signal transduction mechanisms	146	4.33%
U	Intracellular trafficking, secretion, and vesicular transport	45	1.33%
V	Defense mechanisms	62	1.84%

**Table 4:** The potential gene clusters encoding the secondary metabolites in *B. atrophaeus* GQJK17.

Number	Cluster Category^a^	Metabolite^b^	Position	Function	Reference
1	Nrps	Surfactin	BaGK_01865- BaGK_02085	Antifungal, Antibacterial	[15]
2	Bacteriocin-Nrps- Transatpks-Otherks	Bacillaene	BaGK_09425- BaGK_09810	Antibacterial	[35]
3	Transatpks-Nrps	Fengycin	BaGK_10375- BaGK_10710	Antifungal	[15]
4	Ladderane- Cf_fatty_acid -Nrps	Pelgipeptin	BaGK_12700- BaGK_12950	Antibacterial, antifungal	[36]
5	Transatpks	Anthracimycin	BaGK_11000- BaGK_11250	Antibacterial	[37]
6	Nrps-T1pks	Xenocoumacin	BaGK_03970- BaGK_04195	Antifungal	[38]
7	Cf_putative	Bacillomycin	BaGK_20300- BaGK_20325	Antifungal	[39, 40]
8	Sactipeptide- Head_to_tail-Nrps	Rhizocticin	BaGK_01040- BaGK_01305	Antifungal	[41]
9	Nrps	Bacillibactin	BaGK_16720- BaGK_16940	Siderophore	[42]
10	Terpene	Unknown	BaGK_06190 -BaGK_0629 5		
11	Terpene	Unknown	BaGK_10750 -BaGK_1084 5		
12	T3pks	Unknown	BaGK_11290 -BaGK_1152 5		
13	Thiopeptide	Unknown	BaGK_17030 -BaGK_1716 5		

^a^Cluster categories were analyzed by antiSMASH (v.4.0.2).

^b^The secondary metabolites were predicted according to the gene clusters.

## Data Availability

The data used to support the findings of this study are available from the corresponding author upon request.

## Supplementary Materials

Supplementary 1 Supplementary S1: the orthogonal test design of* B. atrophaeus* GQJK17 to optimize the medium.

Supplementary 2 Supplementary S2: the genomic islands information of* B. atrophaeus* GQJK17 that analyzed by IslandViewer 4.
