# Supplementary material for: Analysis of the Complete Genome Sequence of Bacillus atrophaeus GQJK17 Reveals Its Biocontrol Characteristics as a Plant Growth-Promoting Rhizobacterium
Source: Biomed Res Int. 2018 Jun 26;2018:9473542. doi: 10.1155/2018/9473542 (PMC6038694; doi:10.1155/2018/9473542)
Supplement: Supplementary 1 — Supplementary S1: the orthogonal test design of B. atrophaeus GQJK17 to optimize the medium. [file 9473542.f1.doc]

Supplementary S1: Orthogonal test design of GQJK17

| Level | factor | | | |
| --- | --- | --- | --- | --- |
|  | Glucose (%) | soybean meal (%) | NH_4_NO_3_ (%) | MgSO_4_ (%) |
| 1 | 5 | 2 | 0.5 | 0.5 |
| 2 | 3 | 1.5 | 0.3 | 0.3 |
| 3 | 1 | 1 | 0.1 | 0.1 |
