# Supplementary material for: Analysis of the Complete Genome Sequence of Bacillus atrophaeus GQJK17 Reveals Its Biocontrol Characteristics as a Plant Growth-Promoting Rhizobacterium
Source: Biomed Res Int. 2018 Jun 26;2018:9473542. doi: 10.1155/2018/9473542 (PMC6038694; doi:10.1155/2018/9473542)
Supplement: Supplementary 2 — Supplementary S2: the genomic islands information of B. atrophaeus GQJK17 that analyzed by IslandViewer 4. [file 9473542.f2.doc]

Supplementary S2: Genomic islands information of *B. atrophaeus* GQJK17

| GI Number | Start | End | Size | Number of functional genes | Number of unknown genes | GC% |
| --- | --- | --- | --- | --- | --- | --- |
| 1 | 680,876 | 687,967 | 7,091 | 4 | 11 | 43% |
| 2 | 701,688 | 714,779 | 13,091 | 8 | 4 | 43% |
| 3 | 823,317 | 830,290 | 6,973 | 2 | 0 | 47% |
| 4 | 847,660 | 854,551 | 6,891 | 5 | 2 | 35% |
| 5 | 2,017,603 | 2,025,713 | 8,110 | 11 | 1 | 37% |
| 6 | 2,293,560 | 2,300,154 | 6,594 | 4 | 0 | 39% |
| 7 | 2,301,237 | 2,309,917 | 8,680 | 1 | 0 | 39% |
| 8 | 2,338,893 | 2,350,714 | 11,821 | 5 | 5 | 36% |
| 9 | 2,509,569 | 2,517,386 | 7,817 | 7 | 0 | 37% |
| 10 | 2,511,558 | 2,577,617 | 66,059 | 27 | 55 | 39% |
| 11 | 2,570,947 | 2,575,754 | 4,807 | 1 | 7 | 37% |
| 12 | 2,626,627 | 2,644,192 | 17,565 | 3 | 2 | 37% |
| 13 | 2,845,230 | 2,859,213 | 13,983 | 14 | 0 | 42% |
| 14 | 4,290,675 | 4,298,104 | 7,429 | 3 | 1 | 38% |
